# SARS-CoV-2 infection as a risk factor for herpesviridae reactivation: consider the potential influence of corticosteroid therapy

**DOI:** 10.1186/s13054-020-03349-9

**Published:** 2020-10-22

**Authors:** Patrick M. Honore, Leonel Barreto Gutierrez, Luc Kugener, Sebastien Redant, Rachid Attou, Andrea Gallerani, David De Bels

**Affiliations:** ICU Department, Centre Hospitalier Universitaire Brugmann-Brugmann University Hospital, Place Van Gehuchtenplein, 4, 1020 Brussels, Belgium

We read with great interest the recent article by Le Balc'h et al. who concluded that their results suggest that SARS-CoV-2 infection could be a risk factor for *Herpesviridae* reactivation [1]. We would like to make some comments. Glucocorticoid treatment increases the risk of opportunistic infection [2]. Infections that can arise during glucocorticoid use, and for which preventative measures can be taken, include reactivation of *Herpesviridae* that can lead to herpetic pneumonia [2]. In the study of Le Balc'h et al. [1], 44% of patients in the *Herpesviridae* reactivation group received corticosteroids, compared to only 20% of patients in the non-reactivation group. Although the difference did not reach statistical significance due to the low number of patients, the difference is still notable and may explain, at least in part, the difference in *Herpesviridae* reactivation between the groups. *Herpesviridae* reactivations occur easily after high-dose steroids and may lead to fatal disease [3]. Possible infectious complications as a result of *Herpesviridae* reactivations should be considered in patients who receive high-dose glucocorticoid treatment amounting to more than 420 mg of steroid over 4 weeks [4]. This is equal to a daily dose of 52.5 mg of prednisolone [4]. The dose of steroid and the duration of treatment are not reported in the Le Balc'h et al. study [1]. Given that a dose of 52.5 mg of prednisolone is equal to 8 mg of dexamethasone, the dose used in the RECOVERY trial (6 mg) [5] may not increase the risk of reactivation of *Herpesviridae*. However, perhaps even the regimen of 6 mg dexamethasone for 10 days that was used in the RECOVERY trial could result in *Herpesviridae* pulmonary reactivation when given in the context of prolonged mechanical ventilation and concomitant bacterial sepsis.

## Data Availability

Not applicable.
